# An Overview of the Cytoskeleton-Associated Role of PDLIM5

**DOI:** 10.3389/fphys.2020.00975

**Published:** 2020-08-07

**Authors:** Xiaolan Huang, Rongmei Qu, Jun Ouyang, Shizhen Zhong, Jingxing Dai

**Affiliations:** Guangdong Provincial Key Laboratory of Medical Biomechanics, Department of Anatomy, School of Basic Medical Sciences, Southern Medical University, Guangzhou, China

**Keywords:** PDZ and LIM domain 5, microfilament, actin, cytoskeleton, cytoskeleton-associated protein

## Abstract

Regenerative medicine represented by stem cell technology has become one of the pillar medical technologies for human disease treatment. Cytoskeleton plays important roles in maintaining cell morphology, bearing external forces, and maintaining the effectiveness of cell internal structure, among which cytoskeleton related proteins are involved in and play an indispensable role in the changes of cytoskeleton. PDLIM5 is a cytoskeleton-related protein that, like other cytoskeletal proteins, acts as a binding protein. PDZ and LIM domain 5 (PDLIM5), also known as ENH (Enigma homolog), is a cytoplasmic protein with a molecular mass of about 63 KDa that consists of a PDZ domain at the N-terminus and three LIM domains at the C-terminus. PDLIM5 binds to the cytoskeleton and membrane proteins through its PDZ domain and interacts with various signaling molecules, including protein kinases and transcription factors, through its LIM domain. As a cytoskeleton-related protein, PDLIM5 plays an important role in regulating cell proliferation, differentiation and cell fate decision in multiple tissues and cell types. In this review, we briefly summarize the state of knowledge on the *PDLIM5* gene, structural properties, and molecular functional mechanisms of the PDLIM5 protein, and its role in cells, tissues, and organ systems, and describe the possible underlying molecular signaling pathways. In the last part of this review, we will focus on discussing the limitations of existing research and the future prospects of PDLIM5 research in turn.

## Introduction

PDZ and LIM domain 5 (also known as ENH, ENH1, L9, and LIM) is a cytoskeleton-related protein that was first discovered by Kuroda et al. (1996), using yeast two-hybrid technology with protein kinase C (PKC) as the bait protein. PDLIM5, which consists of a PDZ domain and one or more LIM domains, is a PDZ-LIM family member whose sequence is highly conserved across species. The proteins of the PDZ-LIM family (Enigma/LMP-1, ENH, ZASP/Cipher, RIL, ALP, and CLP-36) have been suggested to act as linkers to direct LIM binding proteins to the cytoskeleton (Vallenius et al., 2000). PDZ-LIM protein can act as a signal modulator to affect actin dynamics, regulate cell structure, and control gene transcription to promote the assembly of protein complexes. The PDZ-LIM protein family, which function as protein–protein interaction modules, act as scaffolds to bind to filamentous actin-associated proteins, a range of cytoplasmic signaling molecules, and nuclear proteins, allowing this family to carry out diverse functions during development and adulthood (Krcmery et al., 2010).

Several members of the PDZ-LIM proteins family play a regulatory role in the invasion and migration of cancer cells. Dysfunction of the proteins of the PDZ-LIM family is known to affect the maintenance of organ function and weaken the invasion ability and metastatic potential of cancer cells (Bagheri-Yarmand et al., 2006; Tanaka-Okamoto et al., 2009). According to recent studies, PDLIM5 may be involved in the progression of multiple tumor types (Eeckhoute, 2006; Edlund et al., 2012; Heiliger et al., 2012; Li et al., 2015). The important role of PDLIM5 in various organizational systems have led to a deeper understanding of its physiological function. This review aims to summarize the state of knowledge and progress related to PDLIM5 from multidisciplinary perspectives.

## The *PDLIM5* Gene and Its Expression, and the Structure and Distribution of PDLIM5

### The *PDLIM5* Gene

The *PDLIM5* gene is located on the human chromosome 4q22.3, between markers W1-2900 and W1-3273, and spans 18 exons (Ueki et al., 1999; Te Velthuis and Bagowski, 2007). *PDLIM5* can be categorized as long isoforms with three LIM domains and short isoforms without LIM domains according to the presence, or lack of, three LIM domains, and the long and short isoforms each contain 4∼5 subtypes (Cheng et al., 2010; Ito et al., 2012). Mouse PDLIM5 isoform I (mENH-1) encodes a full-length 591-amino-acid protein containing a PDZ domain and three LIM domains; two smaller transcripts, called mouse PDLIM5 isoform 2 and 3 (mENH-2 and mENH-3), encode a 337-amino-acid protein and a 239-amino-acid protein, respectively. Both mouse PDLIM5 isoforms 2 and 3 lack three LIM domains (Niederlander et al., 2004; Zheng et al., 2010). In humans, four PDLIM5 splice isoforms have been identified: one long isoform (ENH1), which contains three LIM domains at its C-terminal and is widely expressed in all tissues; and three short isoforms (ENH2-4), which are mainly expressed in cardiac and skeletal muscle (Kuroda et al., 1996; Ueki et al., 1999). Analysis of human PDLIM5 transcripts showed that three transcripts (hENH-1,-2,-3) were similar to those of mice, while the fourth transcript (hENH-4) encodes a 215-amino-acid protein lacking three LIM domains (Niederlander et al., 2004).

### The Expression, Structure, and Distribution of PDLIM5

The PDLIM5 protein, also known as ENH, is a member of the Enigma family, which consists of an N-terminal PDZ domain and three C-terminal LIM domains. The main function of PDZ and LIM domains is to participate in protein-protein interactions (Table 1). The PDZ domain, one of the most common protein-protein binding domains, is characterized by a highly conserved 80-90 amino acid sequence, consisting of six anti-parallel β-strands and two α-helices (Fanning and Anderson, 1999; Sheng and Sala, 2001). The PDZ domain provides a protein-binding interface that facilitates the formation of multi-protein complexes with a variety of partners, including membrane-associated proteins, cytoplasmic signaling proteins, and cytoskeleton proteins (Jelen et al., 2003; Krcmery et al., 2010; Zheng et al., 2010; Ito et al., 2012). The LIM domain is approximately 55 amino acids long, and is characterized by highly conserved and spatially defined cysteine and histidine residues that coordinate the binding of two zinc ions to form two zinc finger-like structures. LIM domains can exist in proteins alone, or in combination with other domains (Dawid et al., 1998; Bach, 2000; Kadrmas and Beckerle, 2004; Te Velthuis and Bagowski, 2007). The LIM domains can combine highly diverse partners, ranging from signaling molecules and actin cytoskeletal components to transcription factors, and it also support cellular functions (Dawid et al., 1998). In particular, the cross-linking with actin cytoskeleton, such as LIM domain protein, can maintain the functional structure of cardiomyocytes by a mechanism involving its own binding and actin filament cross-linking, which plays an important role in the development of heart disease (Hoffmann et al., 2014). In addition, LIM domain proteins have also been reported to be involved in the invasion and metastasis of cancer as components and targets of the cytoskeleton (Hoffmann et al., 2016, 2018).

**TABLE 1:** Binding partners of PDLIM5 and their functions.

**Protein**	**Domain**	**Functions**	**References**
Protein kinase A (PKA)	PDZ	Protein kinase	Lin et al., 2013
α-actinin	PDZ	Sarcomere Z-line protein	Nakagawa et al., 2000; Ito et al., 2013
Myotilin	PDZ	Sarcomere Z-line protein	Ito et al., 2013
L-type calcium channel	PDZ	Calcium channels in myocytes	Maturana et al., 2008
YAP	PDZ	Transcriptional co-activator	Elbediwy et al., 2018
Protein kinase C (PKC)	LIM	Protein kinase	Kuroda et al., 1996; Maturana et al., 2011
Protein kinase D1 (PKD1)	LIM	Protein kinase	Maturana et al., 2008
CREB	LIM	CAMP related transcription factors	Ito et al., 2015
ID2	LIM	Differentiation inhibitor	Lasorella and Iavarone, 2006; Nakatani et al., 2016
N-type Ca2+ channels	/	N-type calcium channels in nervous system	Maeno-Hikichi et al., 2003
AMP activated protein kinase	/	Protein kinase	Yan et al., 2015; Liu et al., 2017
KAE1	/	Kidney anion exchanger 1	Su et al., 2017

The PDLIM5 splicing isoforms exhibit tissue-specific expression: the long isoforms are widely expressed in various tissues, while the short isoforms are only highly expressed in cardiac and skeletal muscle (Yamazaki et al., 2010). This differential expression of PDLIM5 may be related to their different roles in different tissues and organ systems (Table 2).

**TABLE 2:** List of different disease involved in PDLIM5.

**Related signaling pathways**	**Disease and development**	**References**
RAS-ERK	Papillary thyroid carcinoma	Wei et al., 2018
AMPK	Prostate cancer	Koutros et al., 2013; Liu et al., 2017
/	Gastric cancer	Li et al., 2015
/	non-small cell carcinoma	Edlund et al., 2012
/	Cancer associated with steroid use	Wang et al., 2016
PKC-β	Heart development	Nakagawa et al., 2000
MicroRNA-17∼92	Cardiomyocyte hypertrophy	Yamazaki et al., 2010; Bang et al., 2014
TGF-β/Smad	Pulmonary hypertension	Chen et al., 2015; Cheng et al., 2016
/	Dilated Cardiomyopathy	Cheng et al., 2010
/	Bipolar disorder	Zhao et al., 2009
/	Depressive disorder	Liu et al., 2008
/	Schizophrenia	Numata et al., 2007
/	Alcohol dependence, type 2 diabetes, and hypertension	Owusu et al., 2017

## The Differential Roles of PDLIM5 in Various Organ Systems

### The Role of PDLIM5 in the Nervous System

PDZ and LIM domain 5 is widely expressed in different regions of the brain, such as the hippocampus, thalamus, hypothalamus, cerebral cortex, and amygdala (Maeno-Hikichi et al., 2003). In central neurons, PDLIM5 is mainly localized in the membrane and cytoplasm, where it regulates neuronal calcium signaling and co-localizes with neurotransmitter-protruding vesicles (Numata et al., 2007); these observations indicate that PDLIM5 plays a role in brain development. Some studies have shown that the expression of PDLIM5 is associated with multiple mental disorders such as bipolar disorder, major depression, and susceptibility to schizophrenia (Liu et al., 2008; Zhao et al., 2009; Herrick et al., 2010; Wang et al., 2016).

In the nervous system, PDLIM5 plays an important role in the formation of nerve growth cones and promotes the differentiation of nerve cells. Some studies have shown that PDLIM5 expression is up-regulated during neural differentiation, and it has been shown that its ectopic expression in neuroblastoma cells leads to the translocation of ID2, which is one of the four members of the ID protein family, called a differentiation inhibitor, from the nucleus to the cytoplasm, resulting in the inactivation of transcriptional and cell-cycle-promoting functions of the latter (Lasorella and Iavarone, 2006). Furthermore, PDLIM5 can form large complexes with PKC and N-type Ca2+ channels to promote the regulation of N-type calcium channel activity (Maeno-Hikichi et al., 2003). Ren et al. (2015) showed that PDLIM5 and PKCε co-exist in the nerve growth cone. Through interaction with α-actinin, PDLIM5 may be involved in regulating microfilament remodeling in neurons and the formation of the PDLIM5-PKCε complex in the nerve growth cone, which acts as a scaffold to mediate PKCε translocation to the membrane during PMA-induced growth cone collapse. It is suggested that PDLIM5 participates in a variety of functions of the nervous system, as well as in a signaling pathway involving the sequestration of the transcription factor ID2 in the cytoplasm. However, the precise mechanisms by which PDLIM5 regulates the functions of the nervous system via ID2 blockade requires further elucidation.

### PDLIM5 in the Heart and Skeletal Muscle

#### Physiological Roles of PDLIM5 in the Heart

The heart undergoes development and begins to function in the early stages of embryonic development. PDLIM5 is expressed at high levels in the skeletal muscle and myocardium, and is considered to be a heart- and skeletal-muscle-specific scaffold protein to regulates mouse heart development (Mu et al., 2015). PDLIM5 is capable of binding to α-actinin through the PDZ domain and co-localizing in the z-disk region of cardiomyocytes, indicating that this protein plays a role in cardiac development. Studies have shown that PDLIM5 mRNAs are mainly expressed in the heart and skeletal muscle of adult rats, and that PDLIM5 acts as a scaffold protein to mediate the transmission of PKCβ signals in cardiomyocytes, playing an important role in development of the muscle cell in an early developmental stage (Nakagawa et al., 2000; Zheng et al., 2010). The PDZ-LIM protein family is highly expressed in the striated muscle. PDLIM5 is similar to other PDZ-LIM members in the striated muscle, in which the PDZ domain binds to α-actinin while the LIM domain binds to several protein kinases C and protein kinase D (Kuroda et al., 1996; Nakagawa et al., 2000). For example, PDLIM5 traditionally activates the PKC through the direct binding of its LIM domain (Maturana et al., 2011) and interacts to PKA (Lin et al., 2013). Transcription factor CREB, which is one of the first transcription factors activated by neurohumoral factors stimulation, is a transcription factor cAMP response element binding protein, is a known target of the PKC and protein kinase D1 (PKD1) pathways (Thonberg et al., 2002; Ozgen et al., 2008). The interaction between PDLIM5 isoform 1 and CREB is necessary for the phosphorylation of CREB at the amino-acid residue Ser133, which promotes the transcriptional activation and nuclear localization of CREB, the phosphorylated CREB enters the cardiomyocyte nucleus to play the role of transcription factor and promote the growth of cardiomyocytes (Ito et al., 2015). Moreover, in neonatal rat cardiomyocytes, PDLIM5 interacts with PKD1 through its LIM domain and forms complexes with PKD1 and cardiac L-type voltage-gated calcium channelα1C subunits to regulate the activity of L-type voltage-gated calcium channels (Maturana et al., 2008). Although the formation of protein complexes such as PDLIM5/PKC/PKD1 is well understood, the downstream molecular events remain to be elucidated.

These results suggest that the localization of PDLIM5 at some subcellular sites and its ability to interact with multiple functional proteins play an important role in cellular and physiological functions. Furthermore, the role of PDLIM5 in the heart was studied using a heart-specific PDLIM5-knockout mouse model. It was found that the ablation of PDLIM5 disrupted the stability of the PDLIM5-Ciphers-Calsarcin complex formed in the z-disk region, thus interfering with the connection between adjacent sarcomeres and extracellular matrix. These effects were found to result in the loss of optimal force transmission and a significant decrease in cardiac shortening fraction, leading to dilated cardiomyopathy (Cheng et al., 2010). Novel polymorphisms in the *PDLIM5* gene encoding the Z-line protein have also been shown to increase the risk of idiopathic dilated cardiomyopathy (Wang et al., 2019).

PDZ and LIM domain 5 is additionally involved in skeletal muscle development. Myogenesis is an important biological process that underpins skeletal muscle regeneration and postnatal growth. The silencing of PDLIM5 increases the nuclear accumulation of differentiation inhibitor (Id2), which inhibits the proliferation and differentiation of myoblasts (Nakatani et al., 2016; Qiu et al., 2016). In addition, the differentiation of, and morphological changes in, skeletal muscle is regulated by a group of transcription factors known as myogenic regulators. PDLIM5 isoform 1 overexpression leads to the up-regulation of MyoD and myogenin, while PDLIM5 isoform 1 knockout significantly decreases the expression of these two proteins; these findings indicate that the main effect of PDLIM5 isoform 1 on muscle cells is to stimulate the transcription of MyoD- and/or myogenin-encoding genes (Ito et al., 2013).

Although the main role of PDLIM5 is as a specific scaffold protein, which to bridge the connection between cytoskeleton and membrane proteins and promote the formation of protein complexes, it is capable of generating numerous splicing isoforms (ENH2-4) that exert various effects on the development of heart and skeletal muscle. In vitro, PDLIM5 isoform 1 has been shown to promote the expression of myogenic genes and myotube formation, while the short PDLIM5 isoform 4 has been found to abrogate myotube-like morphological changes without altering the expression of the myogenic transcription factors MyoD and myogenin (Ito et al., 2013). Furthermore, the overexpression of PDLIM5 isoform 1 prevented ventricular cardiomyocyte hypertrophy induced by vascular stress hormones (Yamazaki et al., 2010). Western blotting analysis of muscle tissue using a non-isoform-specific anti-PDLIM5 antibody showed that PDLIM5 isoform 4 is only expressed in skeletal muscle, with a specific distribution of PDLIM5 members between skeletal muscle and myocardium (Niederlander et al., 2004).

These results suggest that PDLIM5 plays a key role in the development of the myocardium and skeletal muscle. However, the signal transduction mechanisms underlying the role of PDLIM5 in heart and skeletal muscle remain to be further studied. For example, the specific molecular mechanisms by which PDLIM5 regulates the development of myocardium and skeletal muscle through binding protein kinases are unknown. Furthermore, the mechanisms by which PDLIM5 sequesters nuclear protein Id2 in the cytoplasm remain to be elucidated.

#### The Role of PDLIM5 in Cardiovascular Diseases

PDZ and LIM domain 5 is mainly distributed on the z-line of the sarcomere of cardiomyocytes, therefore, the effect of PDLIM5 on myocytes may be related to the contractile function of these cells. PDLIM5-knockout mice exhibit dilated cardiomyopathy, which is characterized by thinning of the left ventricular wall, enlargement of the left ventricular cavity, impaired cardiac contraction, and reduced ejection function (Cheng et al., 2010). PDLIM5 can regulates vascular remodeling, which can as a new pro-atherosclerotic factor to be a therapeutically targeted (Huang et al., 2020). Cardiac remodeling, which is indicative of progression in many cardiovascular diseases, is characterized by cardiomyocyte hypertrophy and myocardial fibrosis, which lead to heart failure (Swynghedauw, 1999; Barry and Townsend, 2010). microRNA (miR-21) derived from cardiac fibroblasts exosomes is a strong paracrine RNA molecule that induces cardiomyocyte hypertrophy (Thum et al., 2008). Recently, it has been reported that PDLIM5 is the direct target of miR-17∼92 (Bang et al., 2014; Chen et al., 2015, 2018). By acting on its target gene *PDLIM5*, miR-21 participates in the interaction between cardiac fibroblasts and cardiomyocytes, thus inducing myocardial pathological hypertrophy (Bang et al., 2014). PDLIM5 also plays a role in vascular smooth muscle. AMP-activated protein kinase is an intracellular energy receptor of (AMPK), which is activated under hypoxia, ischemia, glucose loss, and stress (Steinberg and Kemp, 2009). AMPK is generally considered to be an energy sensor kinase and requires AMP for its activation (Carling et al., 2011). Nakano et al. reported that AMPK controls microtubule dynamics by phosphorylating cytoplasmic connexin-170 (CLIP-170), thus regulating cell migration (Nakano et al., 2010). In addition, AMPK is involved in the regulation of actin cytoskeleton dynamics and plasma membrane reorganization (Bae et al., 2011; Kondratowicz et al., 2013). Studies have shown that AMPK activation plays an important role in neovascularization and metastasis (Hoyer-Hansen and Jaattela, 2007). In vascular smooth muscle cells, AMPK phosphorylates PDLIM5 at Ser177, inhibiting the downstream Rac1-Arp2/3 signaling pathway to mediate cell migration (Yan et al., 2015). In pulmonary artery smooth muscle cells (PASMCs), SMC-specific knockout of PDLIM5 enhances hypoxia-mediated vascular remodeling, while overexpression of PDLIM5 inhibits the TGF-β/Smad signal pathway and prevents hypoxia-induced pulmonary hypertension elevation in vivo (Cheng et al., 2016). In addition, PDLIM5 silencing induces the activity of TGF-β3, TβR1, and TGF-β and increases the overall expression level of Smad2. The suppression of PDLIM5 has been found to enhance the nuclear staining of Samd2/3 (Chen et al., 2015), indicating that PDLIM5 participates in the development of cardiovascular disease by negatively regulating the TGF-β3/Smad2/3 signal pathway. Additionally demonstrated that PDLIM5 plays an important role in the cardiovascular system through miR-mediated regulation of the phenotype of pulmonary artery smooth muscle cells: miR-17∼92 regulates the differentiation of PASMCs through its target PDLIM5, indicating that the miR-17∼92/PDLIM5/TGF-β/Smad pathway is essential for vascular remodeling during the development of pulmonary hypertension. PDLIM5 therefore represents a promising therapeutic target for future cardiovascular drug discovery efforts.

### The Role of PDLIM5 in Tumor

As an actin adaptor protein, PDLIM5 is not only involved in cytoskeletal tissue, cellular processes, and organ development, but is also considered to play roles in tumorigenesis and development (Eeckhoute, 2006; Edlund et al., 2012; Heiliger et al., 2012; Li et al., 2015). PDLIM5 is expressed in many cancer cell lines. In a study of neurologic tumor, it was found that the transcription factor ID2 binds to the PDLIM5 LIM domains, and, in these cancer cells, high levels of PDLIM5 sequester ID2 in the cytoplasm, preventing neuronal differentiation and promoting cell proliferation (Lasorella and Iavarone, 2006).

PDZ and LIM domain 5 is additionally up-regulated in papillary thyroid carcinoma (PTC) tissues; elevated PDLIM5 expression promotes the migration, invasion, and proliferation of PTC cells by activating the RAS-ERK pathway (Wei et al., 2018). PDLIM5 may therefore serve as a therapeutic target in a variety of cancers. It can promote the invasion and metastasis of cancer cells. Genotyping chip detection experiments have shown that *PDLIM5* is overexpressed in prostate cancer tissue (Koutros et al., 2013), and some studies have proved that the utility of serum and urine PDLIM5 levels as indicators for auxiliary diagnosis of prostate cancer, with potential value in predicting the risk of progression in advanced prostate cancer (PCA) (Ma et al., 2014). PDLIM5 plays a key role in regulating the proliferation, invasion, and migration of malignant tumor cells by binding to AMPK and regulating its activation and degradation (Liu et al., 2017). *PDLIM5* may therefore act as an oncogene in the development and progression of PCA.

In view of the important role of PDLIM5 in cancer, some studies have indicated that it is involved in the growth of gastric cancer cells, and suggested that PDLIM5 silencing through the use of small interfering RNAs (si-RNA) may be a potential strategy for the treatment of gastric cancer (Li et al., 2015). In addition, Edlund et al. found that the increased expression of PDLIM5 is related to high tumor proliferation rates in non-small cell carcinoma (Edlund et al., 2012). Steroids such as corticosteroid medications play an important role in the development of cancer; it has been reported that 25 single-nucleotide polymorphisms (SNPs) in *PDLIM5* interact with steroids, thus affecting the occurrence and development of cancer (Wang et al., 2016).

The above findings show that PDLIM5 plays an important role in the occurrence and development of cancer, and that *PDLIM5* represents a candidate oncogene in various cancers.

### The Role of PDLIM5 in Other Diseases

In addition to playing a role in the diseases reported above, PDLIM5 is involved in the link between alcohol dependence and diabetes. Owusu et al. (2017) found that PDLIM5 gene polymorphism is associated alcohol-dependent (AD), type 2 diabetes (T2D), and hypertension, and Several genetic variants of the PDLIM5 gene can affect AD, T2D and hypertension, indicating that PDLIM5 is a shared gene among the three diseases. Therefore, elucidation of the underlying molecular mechanisms and identification of hitherto undiscovered molecular functions of PDLIM5 are expected to enable the development of effective clinical therapies for these diseases.

In addition, PDLIM5 plays a role in the formation of cell extensions. Being a scaffold protein, PDLIM5 is involved in promoting the activity of microfilament-associated proteins. Microfilament-associated proteins play a central role in the process of cell extension (Lanier et al., 2015). Some studies have found that PDLIM5 recruitment to cell extensions, and is necessary to form these extensions, and that PDLIM5 knockout reduces the assembly of actin filaments in cell extensions (Yuda et al., 2018).

## The Relationship Between PDLIM5 and Integrins, and Its Potential Role in the Regulation of Stem Cell Differentiation

Some studies have shown that the PDZ domain of PDZ-LIM protein binds to α-actinin protein at the adhesion junction, which is the site of integrin localization (Xia et al., 1997; Pomies et al., 1999; Vallenius et al., 2000; Tamura et al., 2007). The functional interaction with integrin indicates that PDZ-LIM protein can participate in the adhesion signal cascade, which transmits extracellular signals via intracellular regulatory pathways, thereby modifying the actin cytoskeleton. These findings show that the PDZ-LIM protein plays an overall role in cell–cell and cell–matrix interaction and cell migration (Krcmery et al., 2010). The regulation of PDZ-LIM activity plays an important role in preventing uncontrolled actin recombination, proliferation, and cell migration. For example, PDLIM5 can also bind to the integrin protein kinase (ILK), acting as a scaffold bridge between renal ion exchanger 1 (KAE1) and ILK, providing a bridge between KAE1 and potential microfilaments (Su et al., 2017).

It has been reported that PDLIM5 is recruited from the cytoplasm to the integrin adhesion and F-actin stress fibers and responds to stress by directly binding to the key stress fiber component α-actinin. Microfilaments control the nuclear and cytoplasmic localization of transcriptional co-activators YAP and TAZ to regulate gene expression and mediate the differentiation of MSCs (Dupont et al., 2011; Halder et al., 2012). The effective domains of some proteins that are recruited into the actin structure in a force-dependent manner through the LIM domain regulate actin signal transduction (Smith et al., 2014). The action of mechanical force on integrin results in the recruitment of PDLIM5 to activate the YAP pathway during mechanical transduction (Elbediwy et al., 2018); PDLIM5, a components of the integrin adhesion complex, mediates the relationship between integrin and the cytoskeleton (Horton et al., 2016a, b). Proteomic analysis of integrin-related complexes from MSCs has demonstrated the formation of significant amounts of a vinculin-positive adhesion complex on a hard substrate coated with fibronectin (FN) in MSCs, a subset of which colocalized with, or closely to, clusters of PDLIM1 or PDLIM5 (Ajeian et al., 2016).

PDZ and LIM domain 5 undergoes tension-dependent relocalization in cells. Both embryonic and induced pluripotent stem cells can differentiate into derivatives of the third germ layer; as a result, human pluripotent cells are important tools in regenerative medicine (Thomson et al., 1998; Takahashi and Yamanaka, 2006). Studies have reported that during cardiogenesis, embryonic stem cells exhibit dramatic changes in the expression of metabolic enzymes and cytoskeleton proteins (Konze et al., 2017); in particular, Z-disk related proteins with PDZ and LIM domain proteins, including PDLIM5, are up-regulated during cardiogenesis.

Furthermore, the expression of NKX2.5, an important myocardial transcription factor, results in the generation of specific PDLIM5 splicing variants during the early development of cardiomyocytes, which in turn affects the myogenic differentiation of myocardium (Konze et al., 2017). At present, there are few studies on PDLIM5 in stem cells, elucidation of its mechanism in different stem cell types is warranted to identify its functions in these cells.

## Problems and Perspectives

In this article, we briefly reviewed the known functions of the PDLIM5 protein, the progress in elucidation of its roles in various cellular and physiological processes, and the signaling pathways in which it participates. As a member of the PDZ-LIM protein family, PDLIM5 is involved in actin binding, α-actinin binding, protein kinase binding, acts as a scaffold bridge between connective proteins, and plays an indispensable role in various cellular processes. However, so far, the specific molecular mechanisms underlying the functions of PDLIM5 remain unclear.

Future research directions in investigation of PDLIM5 should seek to answer the following questions:

First, research on PDLIM5 has mainly focused on its roles in tumor, the nervous system, and the cardiovascular system. As a microfilament-associated protein, does PDLIM5 play the same role in other physiological systems or cell lines? Is the *PDLIM5* gene expressed in multiple systems?

Second, although the role of PDLIM5 in diseases has been studied, e.g., its expression is up-regulated during tumor development, the specific mechanisms by which it exerts these effects are unknown, and further elucidation of the underlying mechanisms and other functions is warranted.

Third, PDLIM5 has four splicing isoforms, which perform different functions: what are the mechanisms by which they play different roles? Are these differences in their roles attributable to the presence or absence of LIM domains? Additionally, what is the mechanism by which they play different roles?

Fourth, in regard to strategies used for the inhibition of PDLIM5 expression, only viral transfection has been reported to date; no pharmaceutical compounds that inhibit PDLIM5 expression have been identified. Therefore, additional research on PDLIM5 inhibitors is also critical.

Finally, although PDLIM5 plays a crucial role in binding actin and has attracted much attention as a connecting protein, there are few studies on its effects on the actin cytoskeleton or other cytoskeleton, such as binding to cytoskeleton-related proteins bridging the connection with the cytoskeleton. Does PDLIM5 affect the shape and location of the cytoskeleton in the process of participating in the biological functions of cell?

The effects of PDLIM5-mediated modulation of the cytoskeleton on cell differentiation, proliferation, and other cellular functions remain to be explored in detail in future studies.

## Author Contributions

All authors participated in the conception and writing of the manuscript. SZ, JO, and JD reviewed and suggested modifications to the content. JD designed the structure of the review.

## Conflict of Interest

The authors declare that the research was conducted in the absence of any commercial or financial relationships that could be construed as a potential conflict of interest.
